# Projected Health and Economic Impacts of Achieving the Recommended Dairy Intake in Japan: A Simulation Study of Increased Milk Consumption for Stroke Prevention

**DOI:** 10.3390/nu18060906

**Published:** 2026-03-12

**Authors:** Ryota Wakayama, Michihiro Araki, Mieko Nakamura, Nayu Ikeda

**Affiliations:** 1 National Institute of Health and Nutrition, National Institutes of Biomedical Innovation, Health and Nutrition, 3-17 Senriokashimmachi, Settsu 566-0002, Osaka, Japan; 2 Meiji Co., Ltd., 2-2-1 Kyobashi, Chuo-ku 104-8306, Tokyo, Japan; 3 Department of Pharmaceutical Sciences, College of Pharmaceutical Sciences, Ritsumeikan University, 1-1-1 Noji-higashi, Kusatsu 525-8577, Shiga, Japan

**Keywords:** simulation, dairy, milk, stroke, health economic impact

## Abstract

**Background/Objectives:** Milk consumption is inversely associated with stroke risk. However, the average dairy consumption in Japan is below recommended guidelines. Therefore, we aimed to evaluate potential health and economic impacts of increased milk intake to achieve the recommended daily dairy intake for stroke prevention. **Methods:** A Markov model stratified by sex and age group simulated the effects of achieving the recommended dairy intake—by increasing milk consumption to 180 g/day—on stroke incidence, stroke-related deaths, and national healthcare expenditures among Japanese adults aged 30–79 years over 10 years. Two scenarios were defined; an immediate increase (Scenario 1) and a constant annual growth rate (Scenario 2) in milk intake, whereas the average dairy product consumption in 2023 was maintained in the base-case scenario. **Results:** Compared with the base-case scenario, increasing milk consumption to 180 g/day was projected to reduce stroke incidence and stroke-related deaths by 7.0% in Scenario 1 and by 3.2% in Scenario 2. National healthcare expenditures for stroke were decreased by 5.1% in Scenario 1 and 2.2% in Scenario 2. **Conclusions:** Achieving the recommended dairy intake may contribute to reductions in healthcare costs by preventing stroke in Japan.

## 1. Introduction

Stroke is a major public health concern in Japan, and the fourth leading cause of death [1]. The annual national healthcare expenditure (NHE) for stroke, including inpatient and outpatient costs, was estimated at approximately 11.6 billion US dollars (USD) in 2023 [2]. Among modifiable risk factors, diet plays a crucial role in stroke prevention. Excessive sodium intake is associated with hypertension, a major risk factor for stroke [3]. In addition, serum cholesterol levels are associated with ischemic and hemorrhagic stroke [4,5], highlighting the importance of a healthy diet.

A healthy diet helps prevent noncommunicable diseases, including stroke [6]. Dairy products are important components of a balanced diet and are recommended in many national dietary guidelines worldwide [7]. The Japanese Food Guide Spinning Top, Japan’s official dietary guideline, recommends two servings (2 SV) of dairy per day [8]. Dairy consumption is associated with reduced risks of type 2 diabetes [9], obesity [10], and hypertension [11]. Among dairy products, milk has been associated with a reduced stroke risk in several meta-analyses [12,13,14,15]. In Japan, daily milk consumption has also been associated with delayed onset and reduced mortality from stroke [16,17]. The recommendation of 2 SV corresponds to approximately 180 g of milk per day [8,18]. Milk is nutrient-dense food and provides multiple micronutrients that influence stroke risk. It is the main dietary source of calcium in Japan [19], which is inversely associated with hypertension [20] and stroke [21,22,23]. Milk also contains potassium and magnesium, which are associated with a lower stroke risk [18,22]. However, the mean milk and total dairy intake among Japanese adults is 61.8 g/day and 108.3 g/day, respectively [19], indicating that overall dairy consumption in Japan may be insufficient.

Given evidence that milk consumption is associated with reduced stroke risk, increasing milk consumption in Japan may be a practical approach to achieve the recommended dairy intake and reduce healthcare costs by reducing stroke incidence and mortality. However, the potential health and economic impacts of meeting the recommended dairy intake through increased milk consumption remain unclear. Therefore, we aimed to develop a simulation model to estimate the health and economic impacts of increased milk consumption to achieve the recommended daily dairy intake for stroke prevention in the Japanese population.

## 2. Materials and Methods

### 2.1. Modeling Framework

To evaluate the health and economic impacts of achieving the recommended dairy intake through increased milk consumption, we focused on stroke incidence, stroke-related deaths, and NHE in Japan. Based on data availability, the scope of this study was limited to total stroke, including subarachnoid hemorrhage, intracerebral hemorrhage, and cerebral infarction.

We developed a Markov cohort simulation model using TreeAge Pro Healthcare 2024 (TreeAge Software, Williamstown, MA, USA) to model transitions between multiple health states over time [24]. A closed cohort of Japanese individuals aged 30–79 years in 2023 was simulated over a 10-year period (2023–2032), with each cycle representing 1 year. We focused on adults aged ≥30 years because individuals in this age group account for >95% of stroke cases in Japan, whereas the incidence among those aged <30 years is relatively low [25,26]. This simulation period was set to 10 years to minimize the influence of long-term societal changes and ensure the robustness of the results.

**Figure 1:**
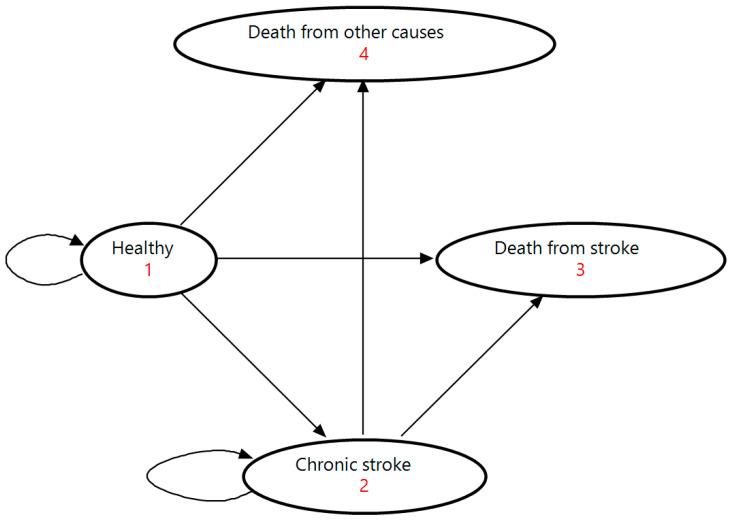
State transition diagram for the Markov model. The model consists of the following four health states: (1) Healthy, (2) Chronic stroke, (3) Death from stroke, and (4) Death from other causes. Straight arrows represent transitions between states, whereas curved arrows indicate remaining in the same state.

In this study, a Markov model stratified by sex and 10-year age group (30–39, 40–49, 50–59, 60–69, and 70–79 years) was constructed. The model included four mutually exclusive health states: “Healthy” “Chronic stroke,” “Death from stroke,” and “Death from other causes” (Figure 1). The “Healthy” state represented individuals who had never experienced a stroke, while the “Chronic stroke” state included those who had survived a stroke. “Death from stroke” referred to individuals who died from stroke, and “Death from other causes” included deaths unrelated to stroke. The acute phase of stroke was not modeled as a separate state but was incorporated into transitions at stroke onset because its duration was shorter than the 1-year simulation cycle.

At the start of the Markov simulation, the Japanese population was stratified into “Healthy” and “Chronic stroke” states based on the prevalence rates of stroke. The number of individuals initially assigned to the “Death from stroke” and “Death from other causes” states was set to zero. During each cycle, individuals in each cohort transitioned between the four health states according to the predefined transition probabilities. Individuals in the “Healthy” state could: (1) remain in the “Healthy” state; (2) experience the first incidence of stroke and survive, transitioning to the “Chronic stroke” state; (3) experience the first incidence of stroke and die during the acute phase (within 28 days), transitioning to the “Death from stroke” state; or (4) die from other causes, transitioning to the “Death from other causes” state. Individuals in the “Chronic stroke” state could: (1) remain in the “Chronic stroke” state without recurrence; (2) experience a recurrent stroke and survive, remaining in the “Chronic stroke” state; (3) experience a recurrent stroke and die during the acute phase, transitioning to the “Death from stroke” state; or (4) die from other causes, transitioning to the “Death from other causes” state. All transitions between the health states were assumed to be irreversible.

### 2.2. Scenarios

In this study, we modeled intervention scenarios in which the recommended dairy intake of 2 SV per day was achieved solely through increased milk consumption. In the guideline, a 180 mL milk bottle was used as a practical example. One serving of dairy contains approximately 100 mg of calcium from milk or dairy products. According to the Standard Tables of Food Composition in Japan (2020 edition), 100 g of milk contains 110 mg of calcium [18]; therefore, 180 g of milk provides approximately 200 mg of calcium, equivalent to the recommended intake of 2 SV. In addition, milk contains other micronutrients relevant to stroke prevention, including potassium and magnesium. Therefore, the target milk intake was set at 180 g/day.

**Figure 2:**
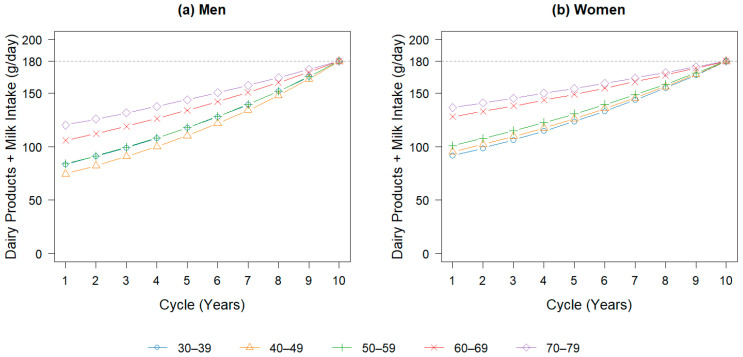
Changes in daily milk intake over 10 years by age group under Scenario 2 in men (**a**) and women (**b**). Each figure illustrates the gradual increase in daily milk intake over a 10-year period for each age-sex group, assuming a constant annual growth rate toward the recommended intake of 180 g/day (Scenario 2). Baseline values correspond to the mean intake of dairy products by age and sex in 2023. The annual growth rates are 108.9%, 110.3%, 108.9%, 106.1%, and 104.6% for men aged 30–39, 40–49, 50–59, 60–69, and 70–79 years, respectively, and 107.8%, 107.4%, 106.6%, 103.9%, and 103.1% for women in the same age groups. The gray dotted line represents the recommended daily intake of dairy products (180 g/day), as specified by the Japanese Food Guide Spinning Top [8].

To reach the target milk intake, two intervention scenarios were defined. In Scenario 1, milk consumption increased immediately to 180 g/day for all age-sex groups. In Scenario 2, milk intake increased gradually over 10 years, reaching 180 g/day by the end of the simulation period for each age-sex group (Figure 2). The base-case scenario assumed that the average dairy consumption by sex and age remained at the 2023 level throughout the simulation period.

Health and economic impacts were evaluated by comparing the projected stroke incidence, stroke-related mortality, and NHE across the base-case and intervention scenarios. In both intervention scenarios, the milk-specific dose-response relative risk for stroke was applied only to the group with incremental milk intake above the baseline average dairy consumption. Net impacts were calculated as the difference between base-case and intervention estimates. Therefore, the estimates reflect only the impacts of increased milk intake and vary according to the baseline gap between current and target dairy intake for each age-sex group.

### 2.3. Input Parameters

For all input parameters, the most recent data available from public databases and peer-reviewed literature as of July 2025 were used as baseline data (Table 1). Age- and sex-specific input parameters are presented in Table 2. For baseline data not available by age group, the 28-day acute fatality rates of stroke were set at 14.9% and 15.7% for men and women, respectively, and recurrence rates were set at 28.2% for men and 24.8% for women [27,28]. The relative risk of stroke per 200 g of milk intake among Asian population was 0.82 (95% confidence interval [CI]: 0.75–0.90) for both sexes [13].

The probability of transitioning between health states was determined using stroke prevalence and incidence rates, stroke-specific and all-cause mortality rates, 28-day acute fatality rates, recurrence rates, and the relative risk of stroke associated with milk intake. The probability of transitioning from the “Healthy” to “Chronic stroke” state was calculated as the product of stroke incidence rates and relative risk associated with milk intake. For individuals in the “Chronic stroke” state, the recurrence rate of stroke was assumed to be constant, regardless of the number of prior events. Similarly, the probability of transitioning from the “Chronic stroke” to “Death from stroke” state was based on a uniform 28-day acute fatality rate, regardless of whether the stroke was an initial or recurrent event. The probability of transitioning from the “Healthy” to “Death from other causes” state was calculated by subtracting stroke mortality rates from all-cause mortality rates.

Stroke-related medical costs included inpatient care, outpatient care, and prescription drug costs (Table 3). Inpatient care costs were assigned to stroke onset events, regardless of whether the stroke was initial or recurrent. Outpatient care and prescription drug costs were assigned to individuals remaining in the “Chronic stroke” state. Prescription drug costs related to stroke were estimated by multiplying the total cardiovascular-related prescription drug costs by the ratio of stroke prevalence to cardiovascular disease prevalence.

NHEs were converted from Japanese yen (JPY) to USD using the average annual exchange rate for 2024 published by the International Monetary Fund (1 USD = 156.65 JPY) [33]. In accordance with economic evaluation guidelines in Japan, 2% annual discount rate was applied to the NHE [34].

### 2.4. Sensitivity Analysis

We performed deterministic one-way sensitivity analyses to examine the impact of parameter uncertainty on the simulation results. The parameters examined included stroke incidence, prevalence, and mortality rates, all-cause mortality rates, the relative risk of stroke associated with milk intake, and the discount rate. Uncertainty in stroke-related parameters was assessed using their respective 95% CIs around the baseline values (Table 2). The discount rate was varied from 0–4%, in accordance with the Japanese economic evaluation guidelines [34].

## 3. Results

### 3.1. Projected Stroke Incidence, Mortality, and NHE Under the Base-Case Scenario

Table 4 presents the projected 10-year cumulative stroke incidence, stroke-related deaths, and NHE under the base-case scenario, assuming the 2023 average daily dairy product intake remained constant. In this scenario, a cumulative total of 1,759,971 stroke cases (2.2% of the total population: 1,096,425 cases among men and 663,546 among women) were projected. Stroke-related deaths were estimated at 267,544 (0.3% of the total population: 163,367 among men and 104,177 among women). The cumulative NHE for stroke was projected to reach USD 51,107,100,072 (USD 30,488,722,609 among men and USD 20,618,377,463 among women). Men exhibited a higher stroke incidence, stroke-related mortality, and NHE than women across all age groups. Projections for stroke cases, stroke-related deaths, and NHE for stroke were highest for the 70–79-year age group for both men and women.

### 3.2. Projected Stroke Cases and Mortality Under Scenarios of Increased Milk Intake to Achieve the Recommended Dairy Intake

Table 5 presents the projected 10-year cumulative numbers of stroke cases and stroke-related deaths preventable by achieving the recommended dairy intake through increased milk consumption, compared with those for the base-case scenario. In Scenario 1, in which milk consumption increased immediately to the target level, 123,618 stroke cases were projected to be prevented over 10 years (a 7.0% reduction; 85,864 among men and 37,754 among women), along with 18,721 stroke-related deaths (a 7.0% reduction; 12,794 among men and 5927 among women). In Scenario 2, in which milk consumption increased gradually each year to reach the target level over 10 years for each age-sex group, 56,480 cases were projected to be prevented (a 3.2% reduction; 38,788 among men and 17,692 among women), along with 8557 stroke-related deaths (a 3.2% reduction; 5779 among men and 2778 among women). In both scenarios, the numbers of prevented stroke cases and deaths were consistently higher for men than for women across all age groups. The highest numbers of prevented stroke cases and deaths were projected in the 70–79-year age group for both sexes, with the largest percentage reductions in the 40–49-year age group among men and the 30–39-year age group among women.

### 3.3. Projected NHE Under Scenarios of Increased Milk Intake to Achieve the Recommended Dairy Intake

Table 6 presents the projected cumulative NHE for stroke that could be saved over 10 years by achieving the recommended dairy intake through increased milk consumption, compared with the base-case scenario. In Scenario 1, the cumulative reduction in NHE was estimated at USD 2,598,230,462, corresponding to a 5.1% reduction (USD 1,742,238,244 for men and USD 855,992,218 for women). In Scenario 2, the cumulative reduction was estimated at USD 1,120,633,534, a 2.2% reduction (USD 741,423,469 for men and USD 379,210,065 for women). In both scenarios, the reduction in NHE was consistently greater among men than among women across all age groups. The largest absolute reduction in NHE was projected in the 70–79-year age group for both sexes, with the highest percentage decrease in the 40–49-year age group.

### 3.4. Sensitivity Analyses

The results of the one-way sensitivity analyses are presented in Table 7. In both scenarios, the largest source of uncertainty in the simulation results was the relative risk of stroke associated with milk intake. The range of cumulative NHE savings due to uncertainty in the relative risk was USD 2,300,333,792 and USD 1,005,552,453 in Scenarios 1 and 2. Conversely, in both scenarios, the smallest sources of uncertainty differed by sex: stroke mortality rates for men and all-cause mortality rates for women.

## 4. Discussion

We investigated the health and economic impacts of achieving the recommended dairy intake—through milk consumption—on stroke in the Japanese population aged 30–79 years. Attaining the recommended dairy intake of 2 SV by increasing milk consumption to 180 g/day was projected to reduce stroke incidence by 2.1–10.6%, stroke-related mortality by 2.1–10.6%, and stroke-related NHE by 1.5–8.5% over a 10-year period compared with the status quo of consumption levels in 2023.

In two intervention scenarios, the largest absolute reduction in NHE for stroke was projected in the 70–79-year age group. However, the percentage reduction in stroke-related NHE was higher in the 30–59-year age groups than in the 60–79-year age groups for both men and women. This pattern may reflect a low baseline dairy product consumption among younger adults, leading to a larger relative impact of increased intake. In contrast, the absolute reduction in NHE was smaller in younger age groups than in older age groups because NHE for stroke increases with age. In addition, in both intervention scenarios, men consistently had higher cumulative incidences of stroke, stroke-related deaths, and NHE than women across all age groups. This difference may be attributable to the higher baseline dairy product intake among women.

Although the Japanese Food Guide Spinning Top recommends 2 SV of total dairy products per day, we focused specifically on milk as the intervention approach. We modeled the achievement of the recommended dairy intake solely through increased milk consumption for two reasons. First, dose-response meta-analyses show that milk is the only dairy subtype with a clear and statistically significant inverse association with stroke risk, particularly in East Asian populations, whereas total dairy, yogurt, and butter show no significant associations and cheese shows only marginal effects [13]. Second, milk accounts for the majority of dairy intake in Japan, making it the most appropriate component for modelling increases in total dairy intake [19]. Therefore, milk was used as the primary approach to achieve the recommended dairy intake in the intervention scenarios.

The beneficial effects of increased milk consumption on stroke can be explained by several mechanisms. Hypertension is a major risk factor for stroke, and a reduction in systolic blood pressure (SBP) of 5 mmHg is associated with a 29% lower risk of stroke [35]. Dairy intake reduces SBP (−0.11 mmHg per 55 g of dairy) [36] and the risk of hypertension (relative risk, 0.96 per 200 g of milk) [37]. In the Japanese population, characterized by relatively high sodium intake and low dairy consumption, dairy intake has been inversely associated with SBP and hypertension risk [38]. Milk is rich in calcium (110 mg per 100 g) [18]. Calcium intake is associated with reduced SBP [39] and hypertension risk (relative risk, 0.93/500 mg) [20] through mechanisms involving parathyroid hormone regulation [40] and vascular structure [41]. Moreover, calcium intake, particularly from dairy products, is inversely associated with stroke risk [21,22,23], including in Asian populations [23]. In addition to calcium, milk contains potassium (150 mg/100 g) and magnesium (10 mg/100 g) [18], which reduce blood pressure [42,43] and are associated with decreased stroke risk [22]. Milk proteins and bioactive peptides also exert antihypertensive effects [44,45], suggesting that these micronutrients jointly contribute to a lower stroke risk.

In addition to blood pressure control, which is mediated by minerals and proteins, saturated fatty acids (SFAs) in milk may also affect stroke risk. Milk contains 2.33 g of SFAs per 100 g [18]. SFAs can increase serum cholesterol levels [46,47], a risk factor for stroke [5], leading the World Health Organization to recommend limiting SFA intake to <10% of total energy intake [48]. Accordingly, the Dietary Reference Intake for Japanese includes dietary goals for SFAs [3], and several Japanese nutrient profiling systems for processed foods classify SFAs as nutrients to limit [49,50,51,52]. However, mean SFA intake among the Japanese population remains <10% of total energy intake [3]; even with milk consumption increased to 180 g/day, SFA intake would not exceed this threshold. Notably, low serum cholesterol levels are inversely associated with stroke risk in Japan [53], and some studies have reported an inverse association between SFA intake and stroke incidence [54,55,56]. Therefore, while excessive milk consumption could increase the intake of nutrients to limit, moderate milk consumption is likely to confer favorable impacts on stroke.

According to the 2023 National Health and Nutrition Survey of Japan, the mean dairy product intake among individuals aged 30–79 years ranged 74.5–136.7 g/day (Table 2), while the mean milk intake ranged 34.8–82.6 g/day [19]. Consistent with these estimates, the milk shipment volume in 2023 totaled 3,211,400 kL, corresponding to an estimated per capita consumption of 71.1 mL/day (73.2 g/day, assuming a milk density of 1.03 g/mL) [57]. The median dairy product intake reported in the same survey ranged 13.5–106.0 g/day for individuals aged 30–79 years, indicating a highly skewed distribution of dairy consumption in Japan, with a substantial proportion of the population consuming little or no milk. When the simulation was conducted under the assumption of median dairy product intake, the maximum potential reduction in stroke-related NHE was estimated to be USD 4,052,321,603, corresponding to a 7.9% reduction compared with the base-case scenario, with reductions of USD 2,693,458,208 and USD 1,358,863,395 for men and women, respectively.

Long-term caregiving costs could not be incorporated into the simulation model owing to insufficient data. Including these costs would likely reveal greater health and economic impacts than those estimated in this study. Stroke is the second leading reason for long-term caregiving in Japan, accounting for 16.1% of all cases requiring caregiving [58]. Stroke onset often limits in activities of daily living and reduces patients’ quality of life [59,60]. Therefore, reducing the risk of stroke among community-dwelling individuals could lower the risk of requiring long-term care and improve quality of life and life satisfaction across the life course. Increased milk consumption is associated with improved cognitive outcomes following stroke-related cognitive decline [61,62], representing another mechanism through which reducing stroke incidence by increasing milk intake may contribute to lowering long-term caregiving expenditures and alleviating the burden on healthcare resources. Milk shipment volumes in Japan have declined over the past decade [57]; however, promoting milk consumption could be a cost-effective strategy for improving public health and curbing NHE in Japan.

Support from policymakers and food manufacturers may be necessary to increase milk consumption among the Japanese population. Based on the shipment volume and total sales (USD 3.86 billion) in 2023 [57], the average retail price of milk is estimated at approximately USD 0.12 per 100 g. Applying this price to the gap between current consumption and the recommended level, the additional cost corresponds to approximately USD 0.05–0.12 per person per day, depending on sex and age group. In this context, promoting higher milk consumption may require a combination of subsidies, nutrition education, and improvements in the food environment.

Incorporating dairy products into the Japanese diet as part of diverse foods may have important implications for improving dietary quality and health economics in Japan [63,64,65]. For instance, adequate yogurt intake has been projected to provide health economic benefits through the prevention of type 2 diabetes [66]. Blood pressure control through dietary salt reduction has been actively promoted as a strategy to prevent cardiovascular diseases under “Health Japan 21 (the third term)” [67]; however, average salt intake has remained stable over the past 10 years [19]. Including milk, which provides potassium, in the diet could reduce the sodium-to-potassium ratio and potentially attenuate the unfavorable effects of high sodium intake. The Japanese Food Guide Spinning Top recommends dairy intake as part of a balanced diet [8], and adherence to this guideline has been associated with improved dietary quality and a lower risk of cerebrovascular disease [68]. Moreover, a new dietary approach, “New Washoku,” which incorporates the umami of dairy products into traditional Japanese diets, has been proposed to promote sodium reduction without compromising taste [69]. Collectively, these approaches suggest that integrating dairy products, particularly milk, into traditional Japanese diets could enhance dietary health while offering potential economic savings in healthcare without sacrificing dietary preferences.

This study had some limitations. First, milk type was not considered in the simulation model because the National Health and Nutrition Survey of Japan does not provide consumption data by type. Based on shipment volume data, 76.6% of milk products in Japan are regular (unmodified) milk [57]. Previous research has indicated that the relative risk of stroke differs by milk type (0.96/200 g for low-fat milk and 1.03/200 g for high-fat milk) [13]. Thus, the estimated health and economic impacts may vary depending on the type of milk consumed. Future research utilizing milk-type-specific data or stratified relative risks will help generate more robust and scientifically detailed estimates. Second, the simulation model did not differentiate between stroke subtypes and applied a uniform relative risk across all subtypes. Previous studies have reported subtype-specific relative risks per 200 g for stroke in global populations: 0.95 (95% CI, 0.89–1.01) for ischemic stroke, 0.90 (95% CI, 0.74–1.09) for hemorrhagic stroke, and 0.88 (95% CI, 0.81–0.96) for fatal stroke [13]. However, subtype-specific data for East Asian populations are limited, and assuming a uniform relative risk may over- or underestimate impacts for specific subtypes. In addition, it may not extend to strokes attributable to specific etiologies that are more common at younger ages in Japan, such as moyamoya disease. Subtype-specific analyses could enhance the precision of future health and economic impact estimates. Third, because our Markov model used population-level relative risks that were not stratified by menopausal or hormonal status, we could not assess whether the protective association of milk consumption with stroke differs in post-menopausal women. Given that hormonal status influences calcium metabolism and vascular physiology, subgroup-specific analyses may provide more precise estimates in future research. Fourth, the simulation did not include the costs required to achieve increased milk consumption. Although the current analysis reflects only potential healthcare savings and does not constitute a full cost-effectiveness evaluation, increasing population-level milk consumption would require programmatic and structural investments, such as policy support and collaboration with policymakers and food manufacturers. These implementation costs could partially offset the savings estimated in our model. Fifth, this study focused on Japanese individuals aged 30–79 years and assumed a 10-year time horizon without accounting for changes in population structure. Therefore, the findings are specific to the assumptions and scope of the analysis. Finally, long-term care expenditures, which are substantial in aging societies, could not be included due to the lack of appropriate data. Incorporating long-term care expenditures in future analyses would allow for a more comprehensive assessment of the societal and economic impacts by achieving the recommended dairy intake through increased milk consumption.

## 5. Conclusions

In this 10-year Markov cohort simulation of Japanese adults aged 30–79 years, increasing milk intake to 180 g/day, corresponding to the recommended dairy intake in Japan, was associated with 7.0% reductions in both stroke incidence and stroke-related mortality, as well as a 5.1% reduction in NHE. Public health institutions may facilitate this through nutrition education, subsidies, and improvements in the food environment. These findings suggest that promoting the recommended dairy intake through milk consumption may help prevent stroke while alleviating the economic burden on the healthcare system. The study provides evidence to inform future public health strategies that emphasize the health and economic advantages of adhering to dietary recommendations.

## Figures and Tables

**Table 1:** Data sources for input parameters for the Markov simulation.

Input Parameters	Data Sources
Total population	Population Estimates, 2024 [29]
Mean dairy product intake (g/day)	National Health and Nutrition Survey in Japan, 2023 [19]
Prevalence rates of stroke	Global Burden of Disease Study 2021 [30]
Incidence rates of stroke	Global Burden of Disease Study 2021 [30]
Mortality rates of stroke	Global Burden of Disease Study 2021 [30]
All-cause mortality rates	Global Burden of Disease Study 2021 [30]
Acute fatality rates of stroke within 28 days	Takashima Cardiovascular Disease Registration System [27]
Recurrence rates of stroke	Shiga Stroke Registry [28]
Relative risk of stroke associated with milk intake	Dose-response meta-analysis of five cohort studies in East Asian countries [13]
National health expenditures for inpatient and outpatient care for stroke	Survey on Medical Insurance Benefits, 2022 [31]
National health expenditures for prescription drugs for stroke	Survey on the Trend of Medical Care Expenditure, 2023 [32]

**Table 2:** Baseline data for input parameters by age and sex for the Markov simulation.

Sex, Age (Years)	Total Population [29]	Mean Dairy Product Intake [19]	Stroke Incidence [30]	Stroke Prevalence [30]	Stroke Mortality [30]	All-Cause Mortality [30]
	No.	g/day	per 100,000	per 100,000	per 100,000	per 100,000
Men						
30–39	6,798,000	83.5	42.4 (30.2–59.1)	426.2 (379.8–479.0)	4.1 (4.0–4.4)	63.4 (63.0–63.8)
40–49	8,303,000	74.5	144.8 (114.5–180.0)	1148.8 (1015.5–1285.2)	15.9 (15.2–16.5)	139.5 (138.4–140.7)
50–59	9,194,000	83.9	270.2 (208.8–335.3)	2860.1 (2543.8–3171.6)	36.7 (35.3–38.1)	378.1 (374.8–381.6)
60–69	7,292,000	105.7	398.9 (306.9–517.4)	5464.0 (4894.2–6027.6)	79.6 (75.6–83.1)	1001.9 (993.7–1010.4)
70–79	7,438,000	120.2	560.9 (424.8–724.5)	8441.3 (7570.3–9391.0)	226.8 (210.5–237.8)	2708.6 (2688.5–2729.5)
Women						
30–39	6,469,000	91.4	26.8 (19.0–38.3)	443.3 (396.6–492.4)	1.8 (1.7–1.9)	36.5 (36.1–36.8)
40–49	8,073,000	95.0	71.8 (55.1–91.6)	846.4 (761.8–934.7)	6.9 (6.6–7.2)	83.9 (83.1–84.7)
50–59	9,084,000	101.0	127.5 (100.1–160.8)	1709.1 (1531.9–1895.0)	14.7 (13.9–15.4)	195.0 (193.1–196.8)
60–69	7,546,000	128.0	218.0 (170.9–276.6)	3136.9 (2845.7–3417.1)	28.1 (25.1–30.1)	424.7 (421.1–428.2)
70–79	8,646,000	136.7	348.1 (270.4–442.4)	5182.2 (4701.0–5708.6)	96.1 (78.4–106.0)	1187.4 (1178.0–1196.6)

Values in parentheses indicate 95% confidence intervals.

**Table 3:** Baseline national healthcare expenditure data for stroke in Japan (US dollars).

Sex, Age (Years)	Inpatient Care [31]	Outpatient Care [31]	Prescription Drugs [32]
Men			
30–39	38,753,773	5,584,819	8,315,682
40–49	179,301,284	21,568,832	29,075,266
50–59	399,335,567	55,544,773	65,120,487
60–69	644,610,740	103,186,945	110,814,964
70–79	1,282,319,379	219,073,369	170,304,477
Women			
30–39	22,116,756	4,338,202	8,299,326
40–49	100,988,631	13,597,236	20,728,388
50–59	226,854,157	31,534,389	38,844,180
60–69	357,676,506	57,217,788	66,258,666
70–79	962,304,709	152,430,800	121,238,509

**Table 4:** Projected 10-year cumulative stroke cases, stroke-related deaths, and national healthcare expenditures under the base-case scenario.

Sex, Age (Years)	Total Population	Incidence		Deaths		National Health Expenditures
	No.	No.	%	No.	%	US Dollars
Men						
30–79	39,025,000	1,096,425	2.8	163,367	0.4	30,488,722,609
30–39	6,798,000	30,765	0.5	4584	0.1	531,362,966
40–49	8,303,000	129,832	1.6	19,345	0.2	2,349,826,649
50–59	9,194,000	259,477	2.8	38,662	0.4	5,113,721,741
60–69	7,292,000	290,769	4.0	43,325	0.6	8,053,183,579
70–79	7,438,000	385,582	5.2	57,452	0.8	14,440,627,674
Women						
30–79	39,818,000	663,546	1.7	104,177	0.3	20,618,377,463
30–39	6,469,000	18,009	0.3	2827	0.0	341,476,045
40–49	8,073,000	60,902	0.8	9562	0.1	1,343,138,697
50–59	9,084,000	120,151	1.3	18,864	0.2	2,899,140,122
60–69	7,546,000	168,055	2.2	26,385	0.3	4,625,519,052
70–79	8,646,000	296,428	3.4	46,539	0.5	11,409,103,548
Both						
30–79	78,843,000	1,759,971	2.2	267,544	0.3	51,107,100,072
30–39	13,267,000	48,774	0.4	7411	0.1	872,839,011
40–49	16,376,000	190,734	1.2	28,907	0.2	3,692,965,346
50–59	18,278,000	379,628	2.1	57,526	0.3	8,012,861,863
60–69	14,838,000	458,824	3.1	69,710	0.5	12,678,702,631
70–79	16,084,000	682,010	4.2	103,991	0.6	25,849,731,222

**Table 5:** Projected cumulative stroke cases and deaths prevented by achieving the recommended dairy intake through an immediate increase in milk consumption (Scenario 1) and a gradual annual increase to the target level (Scenario 2), compared with the base-case scenario over 10 years.

Sex, Age (Years)	Prevented Incidence				Prevented Deaths			
	Scenario 1		Scenario 2		Scenario 1		Scenario 2	
	No.	%	No.	%	No.	%	No.	%
Men								
30–79	85,864	7.8	38,788	3.5	12,794	7.8	5779	3.5
30–39	2974	9.7	1343	4.4	443	9.7	200	4.4
40–49	13,810	10.6	6129	4.7	2058	10.6	913	4.7
50–59	24,804	9.6	11,143	4.3	3696	9.6	1660	4.3
60–69	21,422	7.4	9827	3.4	3192	7.4	1464	3.4
70–79	22,853	5.9	10,346	2.7	3405	5.9	1542	2.7
Women								
30–79	37,754	5.7	17,692	2.7	5927	5.7	2778	2.7
30–39	1568	8.7	717	4.0	246	8.7	112	4.0
40–49	5155	8.5	2369	3.9	809	8.5	372	3.9
50–59	9421	7.8	4356	3.6	1479	7.8	684	3.6
60–69	8754	5.2	4163	2.5	1374	5.2	654	2.5
70–79	12,856	4.3	6087	2.1	2018	4.3	956	2.1
Both Sexes Combined								
30–79	123,618	7.0	56,480	3.2	18,721	7.0	8557	3.2
30–39	4542	9.3	2060	4.2	689	9.3	312	4.2
40–49	18,965	9.9	8498	4.5	2867	9.9	1285	4.4
50–59	34,225	9.0	15,499	4.1	5175	9.0	2344	4.1
60–69	30,176	6.6	13,990	3.0	4566	6.5	2118	3.0
70–79	35,709	5.2	16,433	2.4	5423	5.2	2498	2.4

**Table 6:** Projected cumulative savings of national healthcare expenditures for stroke by achieving the recommended dairy intake through an immediate increase in milk consumption (Scenario 1) and a gradual annual increase to the target level (Scenario 2), compared with the base-case scenario over 10 years.

Sex, Age (Years)	Scenario 1		Scenario 2	
	US Dollars	%	US Dollars	%
Men				
30–79	1,742,238,244	5.7	741,423,469	2.4
30–39	38,785,976	7.3	16,315,123	3.1
40–49	199,014,089	8.5	82,312,362	3.5
50–59	382,604,309	7.5	161,045,929	3.1
60–69	453,707,570	5.6	195,909,499	2.4
70–79	668,126,299	4.6	285,840,556	2.0
Women				
30–79	855,992,218	4.2	379,210,065	1.8
30–39	19,567,855	5.7	8,314,751	2.4
40–49	86,674,332	6.5	37,282,849	2.8
50–59	176,448,756	6.1	76,786,233	2.6
60–69	182,462,822	3.9	81,732,809	1.8
70–79	390,838,452	3.4	175,093,422	1.5
Both Sexes Combined				
30–79	2,598,230,462	5.1	1,120,633,534	2.2
30–39	58,353,831	6.7	24,629,874	2.8
40–49	285,688,421	7.7	119,595,211	3.2
50–59	559,053,065	7.0	237,832,162	3.0
60–69	636,170,392	5.0	277,642,308	2.2
70–79	1,058,964,751	4.1	460,933,978	1.8

**Table 7:** Lower and upper bounds of uncertainty intervals of projected 10-year cumulative savings in national health expenditures for stroke (US dollars) among individuals aged 30–79 years, achieved by meeting the recommended dairy intake through an immediate (Scenario 1) or gradual (Scenario 2) increase in milk consumption.

Sex, Parameters	Scenario 1		Scenario 2	
	Lower Bound	Upper Bound	Lower Bound	Upper Bound
Men				
Relative risk of stroke	942,916,171	2,479,973,134	398,492,304	1,062,330,377
Discount rate	1,599,900,604	1,906,521,369	655,323,772	842,455,145
Incidence rate of stroke	1,696,149,208	1,795,435,617	724,567,926	760,797,229
Prevalence rate of stroke	1,719,279,851	1,769,734,143	732,745,071	751,794,530
Mortality rate of stroke	1,741,651,567	1,742,664,848	741,058,369	741,688,681
All-cause mortality rate	1,741,383,454	1,743,058,414	740,892,505	741,933,037
Women				
Relative risk of stroke	460,955,925	1,224,232,753	203,194,151	544,908,531
Discount rate	785,579,344	937,292,107	335,320,857	430,695,228
Incidence rate of stroke	835,847,059	880,199,392	371,664,343	388,243,339
Prevalence rate of stroke	846,802,202	866,607,996	375,680,688	383,279,650
Mortality rate of stroke	855,653,431	856,187,206	378,993,506	379,334,636
All-cause mortality rate	855,785,709	856,202,615	379,078,459	379,344,159

## Data Availability

The data used in this study are derived from publicly available sources, which are summarized in Table 1.

## References

[1] Ministry of Health, Labour and Welfare (2024). Vital Statistics. https://www.mhlw.go.jp/toukei/list/81-1.html.

[2] Ministry of Health, Labour and Welfare (2023). Estimates of National Medical Care Expenditure. https://www.mhlw.go.jp/toukei/saikin/hw/k-iryohi/23/dl/R05data.pdf.

[3] Ministry of Health, Labour and Welfare, Japan (2025). The Dietary Reference Intakes for Japanese. https://www.mhlw.go.jp/stf/newpage_44138.html.

[4] Xie L., Wu W., Chen J., Tu J., Zhou J., Qi X., Yin X. (2017). Cholesterol levels and hemorrhagic stroke risk in East Asian versus non-East Asian populations: A systematic review and meta-analysis. Neurologist.

[5] Gong X., Chen L., Song B., Han X., Xu W., Wu B., Sheng F., Lou M. (2022). Associations of lipid profiles with the risk of ischemic and hemorrhagic stroke: A systematic review and meta-analysis of pro-spective cohort studies. Front. Cardiovasc. Med..

[6] World Health Organization Healthy Diet. https://www.who.int/news-room/fact-sheets/detail/healthy-diet.

[7] Cámara M., Giner R.M., González-Fandos E., López-García E., Mañes J., Portillo M.P., Rafecas M., Domínguez L., Martínez J.A. (2021). Food-based dietary guidelines around the world: A comparative analysis to update AESAN Scientific Committee dietary recommendations. Nutrients.

[8] Ministry of Health, Labour and Welfare Japanese Food Guide Spinning Top. https://www.mhlw.go.jp/bunya/kenkou/eiyou-syokuji.html.

[9] Gijsbers L., Ding E.L., Malik V.S., de Goede J., Geleijnse J.M., Soedamah-Muthu S.S. (2016). Consumption of dairy foods and diabetes incidence: A dose-response meta-analysis of observational studies. Am. J. Clin. Nutr..

[10] Kim D., Kim J. (2017). Dairy consumption is associated with a lower incidence of the metabolic syndrome in middle-aged and older Korean adults: The Korean Genome and Epidemiology Study (KoGES). Br. J. Nutr..

[11] Wang H., Fox C.S., Troy L.M., McKeown N.M., Jacques P.F. (2015). Longitudinal association of dairy consumption with the changes in blood pressure and the risk of incident hypertension: The Framingham Heart Study. Br. J. Nutr..

[12] Elwood P.C., Givens D.I., Beswick A.D., Fehily A.M., Pickering J.E., Gallacher J. (2008). The survival advantage of milk and dairy consumption: An overview of evidence from cohort studies of vascular diseases, diabetes and cancer. J. Am. Coll. Nutr..

[13] de Goede J., Soedamah-Muthu S.S., Pan A., Gijsbers L., Geleijnse J.M. (2016). Dairy consumption and risk of stroke: A systematic review and updated dose-response meta-analysis of prospective cohort studies. J. Am. Heart Assoc..

[14] Alexander D.D., Bylsma L.C., Vargas A.J., Cohen S.S., Doucette A., Mohamed M., Irvin S.R., Miller P.E., Watson H., Fryzek J.P. (2016). Dairy consumption and CVD: A systematic review and meta-analysis. Br. J. Nutr..

[15] Soedamah-Muthu S.S., de Goede J. (2018). Dairy consumption and cardiometabolic diseases: Systematic review and updated meta-analyses of prospective cohort studies. Curr. Nutr. Rep..

[16] Wang C., Yatsuya H., Lin Y., Sasakabe T., Kawai S., Kikuchi S., Iso H., Tamakoshi A. (2020). A Milk intake and stroke mortality in the Japan collaborative cohort study-a Bayesian survival analysis. Nutrients.

[17] Lu Y., Sugawara Y., Hozawa A., Iwasaki M., Sawada N., Kanehara R., Oze I., Ito H., Miyagawa N., Koyanagi Y.N. (2025). Association between dairy intake and all-cause and cause-specific mortality: A pooled analysis of 0.4 million Japanese adults from 10 population-based cohort studies. Int. J. Epidemiol..

[18] Ministry of Education, Culture, Sports, Science and Technology Standard Tables of Food Composition in Japan -2020- (Eighth Revised Edition). https://www.mext.go.jp/content/20201225-mxt_kagsei-mext_01110_011.pdf.

[19] Ministry of Health, Labour and Welfare The National Health and Nutrition Survey, 2023. https://www.mhlw.go.jp/stf/seisakunitsuite/bunya/kenkou_iryou/kenkou/eiyou/r5-houkoku_00001.html.

[20] Jayedi A., Zargar M.S. (2019). Dietary calcium intake and hypertension risk: A dose-response meta-analysis of prospective cohort studies. Eur. J. Clin. Nutr..

[21] Larsson S.C., Orsini N., Wolk A. (2013). Dietary calcium intake and risk of stroke: A dose-response meta-analysis. Am. J. Clin. Nutr..

[22] Adebamowo S.N., Spiegelman D., Willett W.C., Rexrode K.M. (2015). Association between intakes of magnesium, potassium, and calcium and risk of stroke: 2 cohorts of US women and updated meta-analyses. Am. J. Clin. Nutr..

[23] Wang Z.M., Bu X.X., Zhou B., Li Y.F., Nie Z.L. (2023). Dietary calcium intake and the risk of stroke: Meta-analysis of cohort studies. Nutr. Metab. Cardiovasc. Dis..

[24] TreeAge Software, Inc (2024). TreeAge Pro Healthcare.

[25] Ministry of Health, Labour and Welfare The National Health and Nutrition Survey, 2010. https://www.mhlw.go.jp/bunya/kenkou/eiyou/h22-houkoku.html.

[26] The Japan Stroke Data Bank (2024). Assessment of Stroke Treatment in Our Country Using a Stroke Registry 2024 Report.

[27] Rumana N., Kita Y., Turin T.C., Nakamura Y., Takashima N., Ichikawa M., Sugihara H., Morita Y., Hirose K., Kawakami K. (2014). Acute case-fatality rates of stroke and acute myocardial infarction in a Japanese population: Takashima stroke and AMI registry, 1989–2005. Int. J. Stroke.

[28] Takashima N., Arima H., Kita Y., Fujii T., Miyamatsu N., Komori M., Sugimoto Y., Nagata S., Miura K., Nozaki K. (2017). Incidence, management and short-term outcome of stroke in a general population of 1.4 million Japanese—Shiga Stroke Registry. Circ. J..

[29] Statistics Bureau of Japan Result of the Population Estimates. https://www.stat.go.jp/english/data/jinsui/2.html.

[30] Institute for Health Metrics and Evaluation Global Burden of Disease Study 2021 (GBD 2021) Results. https://vizhub.healthdata.org/gbd-results/.

[31] Ministry of Health, Labour and Welfare (2022). Survey on Medical Insurance Benefits. https://www.e-stat.go.jp/stat-search/files?page=1&layout=datalist&toukei=00450389&tstat=000001044924&cycle=0&tclass1=000001044945&tclass2=000001220980&tclass3val=0.

[32] Ministry of Health, Labour and Welfare Survey on the Trend of Medical Care Expenditures. https://www.mhlw.go.jp/topics/medias/year/23/gaiyou.html.

[33] International Monetary Fund IMF Data Access to Macroeconomic & Financial Data. https://data.imf.org/?sk=388dfa60-1d26-4ade-b505-a05a558d9a42.

[34] Fukuda T., Shiroiwa T., Ikeda S., Igarashi A., Akazawa M., Ishida H., Noto S., Saito S., Sakamaki H., Shimozuma K. (2013). Guideline for economic evaluation of healthcare technologies in Japan. J. Natl. Inst. Public Health.

[35] Thomopoulos C., Parati G., Zanchetti A. (2016). Effects of blood pressure lowering on outcome incidence in hypertension: 7. Effects of more vs. less intensive blood pressure lowering and different achieved blood pressure levels—Updated overview and meta-analyses of randomized trials. J. Hypertens..

[36] Ding M., Huang T., Bergholdt H.K., Nordestgaard B.G., Ellervik C., Qi L., CHARGE Consortium (2017). Dairy consumption, systolic blood pressure, and risk of hypertension: Mendelian randomization study. BMJ.

[37] Soedamah-Muthu S.S., Verberne L.D., Ding E.L., Engberink M.F., Geleijnse J.M. (2012). Dairy consumption and incidence of hypertension: A dose-response meta-analysis of prospective cohort studies. Hypertension.

[38] Kawata D., Ueno H.M., Nakano A., Tatara Y., Tamada Y., Mikami T., Murashita K., Nakaji S., Itoh K. (2025). Dairy consumption has a partial inverse association with systolic blood pressure and hypertension in populations with high salt and low dairy diets: Cross-sectional data analysis from the Iwaki Health Promotion Project. Hypertens. Res..

[39] Cormick G., Ciapponi A., Cafferata M.L., Cormick M.S., Belizán J.M. (2022). Calcium supplementation for prevention of primary hypertension. Cochrane Database Syst. Rev..

[40] Zhang Y., Zhang D.Z. (2018). Circulating parathyroid hormone and risk of hypertension: A meta-analysis. Clin. Chim. Acta.

[41] Villa-Etchegoyen C., Lombarte M., Matamoros N., Belizán J.M., Cormick G. (2019). Mechanisms involved in the relationship between low calcium intake and high blood pressure. Nutrients.

[42] Aburto N.J., Hanson S., Gutierrez H., Hooper L., Elliott P., Cappuccio F.P. (2013). Effect of increased potassium intake on cardiovascular risk factors and disease: Systematic review and meta-analyses. BMJ.

[43] Kass L., Weekes J., Carpenter L. (2012). Effect of magnesium supplementation on blood pressure: A meta-analysis. Eur. J. Clin. Nutr..

[44] Hidayat K., Du H.Z., Yang J., Chen G.C., Zhang Z., Li Z.N., Qin L.Q. (2017). Effects of milk proteins on blood pressure: A meta-analysis of randomized control trials. Hypertens. Res..

[45] Marcone S., Belton O., Fitzgerald D.J. (2017). Milk-derived bioactive peptides and their health promoting effects: A potential role in atherosclerosis. Br. J. Clin. Pharmacol..

[46] Mensink R.P., Katan M.B. (1992). Effect of dietary fatty acids on serum lipids and lipoproteins. A meta-analysis of 27 trials. Arterioscler. Thromb..

[47] Mensink R.P., Zock P.L., Kester A.D., Katan M.B. (2003). Effects of dietary fatty acids and carbohydrates on the ratio of serum total to HDL cholesterol and on serum lipids and apolipoproteins: A meta-analysis of 60 controlled trials. Am. J. Clin. Nutr..

[48] World Health Organization Saturated Fatty Acid and Trans-Fatty Acid Intake for Adults and Children: WHO Guideline. https://www.who.int/publications/i/item/9789240073630.

[49] Wakayama R., Drewnowski A., Horimoto T., Saito Y., Yu T., Suzuki T., Takasugi S. (2024). Development and validation of the Meiji Nutritional Profiling System (Meiji NPS) to address dietary needs of adults and older adults in Japan. Nutrients.

[50] Wakayama R., Drewnowski A., Horimoto T., Yu T., Saito Y., Suzuki T., Honda K., Kanaya S., Takasugi S. (2024). Development and validation of the Meiji Nutritional Profiling System per serving size. Nutrients.

[51] Takebayashi J., Takimoto H., Okada C., Tousen Y., Ishimi Y. (2024). Development of a nutrient profiling model for processed foods in Japan. Nutrients.

[52] Wakayama R., Yu T., Drewnowski A., Takasugi S., Horimoto T., Tsutsumi C. (2025). Development and validation of the Meiji Nutritional Profiling System for children. Front. Nutr..

[53] Cui R., Iso H., Toyoshima H., Date C., Yamamoto A., Kikuchi S., Kondo T., Watanabe Y., Koizumi A., Inaba Y. (2007). Serum total cholesterol levels and risk of mortality from stroke and coronary heart disease in Japanese: The JACC study. Atherosclerosis.

[54] Yamagishi K., Iso H., Kokubo Y., Saito I., Yatsuya H., Ishihara J., Inoue M., Tsugane S., JPHC Study Group (2013). Dietary intake of saturated fatty acids and incident stroke and coronary heart disease in Japanese communities: The JPHC Study. Eur. Heart J..

[55] Muto M., Ezaki O. (2018). High dietary saturated fat is associated with a low risk of intracerebral hemorrhage and ischemic stroke in Japanese but not in non-Japanese: A review and meta-analysis of prospective cohort studies. J. Atheroscler. Thromb..

[56] Kang Z.Q., Yang Y., Xiao B. (2020). Dietary saturated fat intake and risk of stroke: Systematic review and dose-response meta-analysis of prospective cohort studies. Nutr. Metab. Cardiovasc. Dis..

[57] FUJI KEIZAI Co., Ltd (2024). Food Service Industry Marketing Data Book 2025.

[58] Ministry of Health, Labour and Welfare (2022). Comprehensive Survey of Living Conditions. https://www.mhlw.go.jp/toukei/saikin/hw/k-tyosa/k-tyosa22/index.html.

[59] Niemi M.L., Laaksonen R., Kotila M., Waltimo O. (1988). Quality of life 4 years after stroke. Stroke.

[60] Kim E.S., Kim J.W., Kang H.J., Bae K.Y., Kim S.W., Kim J.T., Park M.S., Cho K.H., Kim J.M. (2018). Longitudinal impact of depression on quality of life in stroke patients. Psychiatry Investig..

[61] Wu L., Sun D. (2016). Meta-analysis of milk consumption and the risk of cognitive disorders. Nutrients.

[62] Amanat S., Dordevic A.L., Brodtmann A., Cardoso B.R. (2025). Associations between diet and cognitive function in stroke survivors: A systematic review and meta-analysis. Adv. Nutr..

[63] Saito A., Okada E., Tarui I., Matsumoto M., Takimoto H. (2019). The association between milk and dairy products consumption and nutrient intake adequacy among Japanese adults: Analysis of the 2016 National Health and Nutrition Survey. Nutrients.

[64] Tsugane S. (2021). Why has Japan become the world’s most long-lived country: Insights from a food and nutrition perspective. Eur. J. Clin. Nutr..

[65] Shirota M., Watanabe N., Suzuki M., Kobori M. (2022). Japanese-style diet and cardiovascular disease mortality: A systematic review and meta-analysis of prospective cohort studies. Nutrients.

[66] Wakayama R., Araki M., Nakamura M., Ikeda N. (2025). The cost-effectiveness of increased yogurt intake in type 2 diabetes in Japan. Nutrients.

[67] Ministry of Health, Labour and Welfare Health Japan 21 (the Third Term). https://www.mhlw.go.jp/stf/seisakunitsuite/bunya/kenkou_iryou/kenkou/kenkounippon21_00006.html.

[68] Kurotani K., Akter S., Kashino I., Goto A., Mizoue T., Noda M., Sasazuki S., Sawada N., Tsugane S., Japan Public Health Center based Prospective Study Group (2016). Quality of diet and mortality among Japanese men and women: Japan Public Health Center based prospective study. BMJ.

[69] Ishida H. (2018). Milk, dairy products and bone health. Milk and dairy products and “Washoku”—“New washoku”. Clin. Calcium.

